# Genetic interactions between polycystin-1 and Wwtr1 in osteoblasts define a novel mechanosensing mechanism regulating bone formation in mice

**DOI:** 10.1038/s41413-023-00295-4

**Published:** 2023-10-26

**Authors:** Zhousheng Xiao, Li Cao, Micholas Dean Smith, Hanxuan Li, Wei Li, Jeremy C. Smith, Leigh Darryl Quarles

**Affiliations:** 1 Department of Medicine, University of Tennessee Health Science Center, Memphis, TN 38163 USA; 2 UT/ORNL Center for Molecular Biophysics, Oak Ridge National Laboratory, Oak Ridge, TN 37830 USA; 3 Department of Biochemistry and Cellular and Molecular Biology, The University of Tennessee-Knoxville, Knoxville, TN 37996-1939 USA; 4 Department of Pharmaceutical Sciences, University of Tennessee Health Science Center, Memphis, TN 38163 USA

**Keywords:** Bone, Bone quality and biomechanics

## Abstract

Molecular mechanisms transducing physical forces in the bone microenvironment to regulate bone mass are poorly understood. Here, we used mouse genetics, mechanical loading, and pharmacological approaches to test the possibility that polycystin-1 and Wwtr1 have interdependent mechanosensing functions in osteoblasts. We created and compared the skeletal phenotypes of control *Pkd1*^flox/+^;*Wwtr1*^flox/+^, *Pkd1*^*Oc-cKO*^, *Wwtr1*^*Oc-cKO*^, and *Pkd1/Wwtr1*^*Oc-cKO*^ mice to investigate genetic interactions. Consistent with an interaction between polycystins and Wwtr1 in bone in vivo, *Pkd1/Wwtr1*^*Oc-cKO*^ mice exhibited greater reductions of BMD and periosteal MAR than either *Wwtr1*^*Oc-cKO*^ or *Pkd1*^*Oc-cKO*^ mice. Micro-CT 3D image analysis indicated that the reduction in bone mass was due to greater loss in both trabecular bone volume and cortical bone thickness in *Pkd1*/*Wwtr1*^Oc-cKO^ mice compared to either *Pkd1*^Oc-cKO^ or *Wwtr1*^Oc-cKO^ mice. *Pkd1*/*Wwtr1*^Oc-cKO^ mice also displayed additive reductions in mechanosensing and osteogenic gene expression profiles in bone compared to *Pkd1*^Oc-cKO^ or *Wwtr1*^Oc-cKO^ mice. Moreover, we found that *Pkd1/Wwtr1*^*Oc-cKO*^ mice exhibited impaired responses to tibia mechanical loading in vivo and attenuation of load-induced mechanosensing gene expression compared to control mice. Finally, control mice treated with a small molecule mechanomimetic, MS2 that activates the polycystin complex resulted in marked increases in femoral BMD and periosteal MAR compared to vehicle control. In contrast, *Pkd1*/*Wwtr1*^Oc-cKO^ mice were resistant to the anabolic effects of MS2. These findings suggest that PC1 and Wwtr1 form an anabolic mechanotransduction signaling complex that mediates mechanical loading responses and serves as a potential novel therapeutic target for treating osteoporosis.

## Introduction

In vivo and in vitro studies demonstrate that the polycystin-1(PC1)/polycystin-2 (PC2) heterotetrameric complex functions in osteoblasts and osteocytes to regulate bone mass^1,2^ and acts as a mechanosensor to transduce the bone anabolic response to mechanical loading in vivo.^3,4^ Genetic ablation of either PC1 or PC2 deficiency in osteoblasts or osteocytes has similar effects in reducing bone mass by decreasing osteoblast-mediated bone formation.^3,5–8^ PC1 and PC2 mechanosensing functions are mediated by heterotetrameric complex activation of common signal transduction pathways. In this regard, PC1 and PC2 conditional knockout models exhibit concordant effects in impairing osteoblast-mediated bone formation. However, PC1 and PC2 have discordant effects on bone marrow adipogenesis that implicates separate signaling mechanisms.^4,6,9^ In this regard, PC1 deficiency stimulates adipogenesis, leading to increased bone marrow adipose tissue (MAT) deposition,^2,6^ whereas PC2 loss-of-function inhibits adipogenesis.^4^ Additional in vitro and in vivo data show that PC1 activates Runx2 transcriptional activity to stimulate osteoblastogenesis but diminishes PPARγ signaling leading to reduced bone marrow fat.^2,4^ In agreement with the low turnover bone disorder in *Pkd1* mouse models, not only bone-specific alkaline phosphatase was significantly lower in patients with ADPKD but also histomorphometric parameters of bone formation were decreased compared to control subjects without ADPKD.^10–12^ The molecular mechanisms underlying the different effects of PC1 and PC2 on osteoblastogenesis and adipogenesis are not clear. In the current studies, we sought to understand the mechanism for the apparent PC1-specific effect in reciprocally regulating transcriptional control of osteoblastogenesis and adipogenesis.

The Hippo-Yap/Wwtr1(also called TAZ) pathway is also regulated by mechanical forces.^13–16^ Alterations of matrix stiffness in cell culture modulates nuclear translocation of non-phosphorylated Wwtr1 resulting in co-activation of Runx2 and stimulation of osteoblastogenesis and in Wwtr1 binding to PPARγ to inhibit adipogenesis.^2,17,18^ The physiological importance of Wwtr1 in bone homeostasis is revealed by transgenic overexpression of *Wwtr1* in osteoblasts in mice, which increases osteoblast-mediated bone formation and inhibits bone marrow adipogenesis^19^; depletion of *Wwtr1* in zebrafish, which impairs bone development,^18^ and global knockout of *Wwtr1* in mice, which have small stature and ossification defects.^20^ Based on these observations, we posit that PC1 dependent Wwtr1 signaling might explain the differential functions of PC1 and PC2 on adipogenesis.

Recent studies show crosstalk between PC1 and Wwtr1 signaling that is mediated by the binding of Wwtr1 to the PC1 C-terminal tail.^2,17^ The PC1/Wwtr1 complex is cleaved to allow nuclear translocation of Wwtr1, a mechanism of Wwtr1 regulation that differs from the canonical Hippo regulation of Yap/Wwtr1 signaling.^21^ Wwtr1 binds to the PC1 C-terminal tail (PC1-CTT) and undergoes nuclear translocation in response to changes in bone ECM microenvironment to stimulate osteoblastogenesis and inhibit adipogenesis through transcriptional co-activation of Runx2 and co-repression of PPARγ activity.^2^ Wwtr1 also binds to PC2, leading to PC2 degradation.^22^ These observations in bone parallel the interactions between PC1/PC2 and Wwtr1 in primary cilia in renal epithelial cells.^20,23,24^ In this regard, *Wwtr1* knockout from the kidney result in cystic kidney disease in mice, similar to polycystin complex inactivation, suggesting that PC1/PC2 and *Wwtr1* act through common pathways in the kidney as well as bone.^20,23,24^ Furthermore, a small molecule mechanomimetic (named as molecular staple, MS) that binds to the PC1:PC2 C-terminal tail in a presumptive coiled-coil region (e.g. PC1 residue Tyr^4236^ and PC2 residues Arg^877^, Arg^878^, and Lys^874^) has been shown to activate this complex and mimic the effects of physical forces to activate polycystins/Wwtr1 signaling and stimulate bone mass in mice.^2^ Collectively these observations suggest that Wwtr1 modulates polycystin’s mechanosensing functions to differentially regulate osteoblastogenesis and adipogenesis.

In this study, we examined the interaction between PC1 and Wwtr1 in mouse bone by using *Osteocalcin* (*Oc*)-Cre to conditionally delete both *Pkd1* and *Wwtr1* in osteoblasts. We compared skeletal phenotypes of double *Pkd1/Wwtr1*^*Oc-cKO*^ mice with single conditional *Pkd1* and *Wwtr1* null mice under baseline conditions, after mechanical loading, and following treatment with a more potent mechanomimetic, MS2, that activates the PC1/PC2 complex. We found that genetic ablation of PC1 and Wwtr1 in osteoblasts results in additive loss of bone mass and anabolic responses to mechanical loading. Compound PC1 and Wwtr1 deficient mice were also resistant to the bone inductive effects of the MS2 mechanomimetic in vivo. Our findings provide a new mechanism whereby Wwtr1 regulates skeletal homeostasis through co-dependent functions with PC1 in osteoblasts.

## Results

### *Wwtr1* has gene-dosage dependent effects on bone mass

**Fig. 1:**
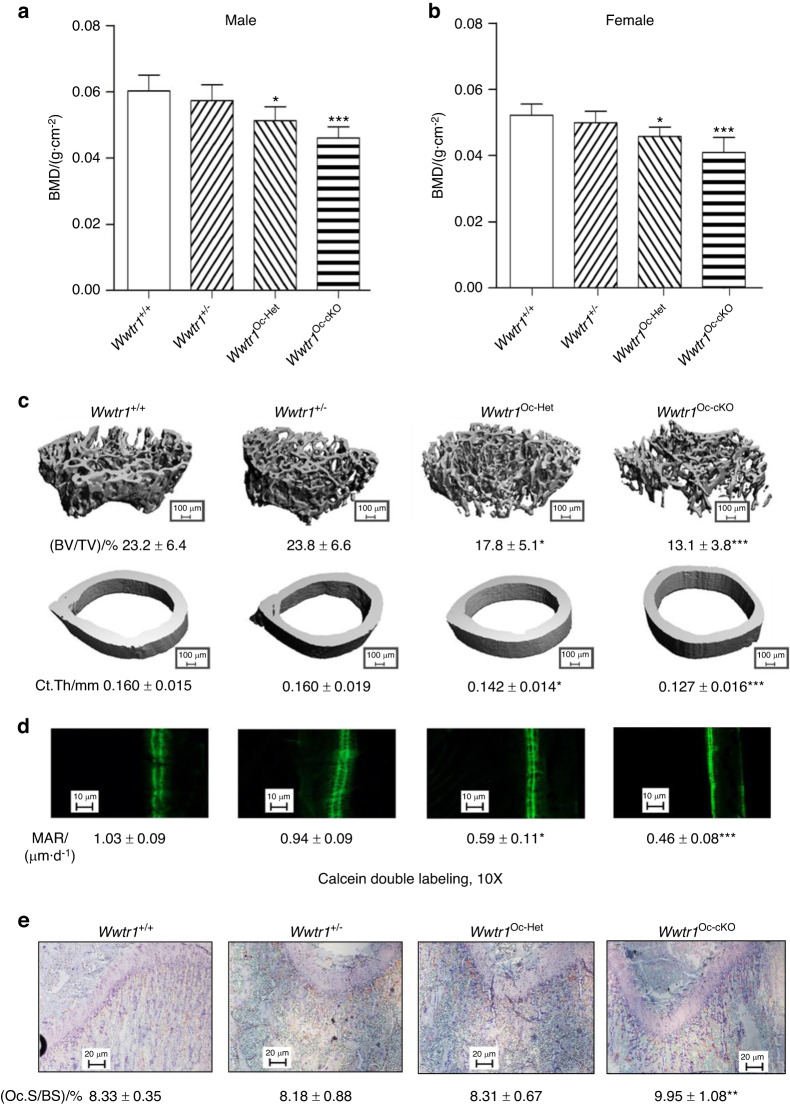
Conditional deletion of *Wwtr1* in mature osteoblasts on postnatal bone homeostasis. **a**, **b** Bone mineral density by DEXA scan in male and female mice. **c** Bone structure by micro-CT 3D images analysis from both male and female mice. **d** Periosteal mineral apposition rate (MAR) by calcein double labeling. There was a significant reduction in periosteal MAR in single *Wwtr1*^Oc-Het^ heterozygous mice compared with age-matched control mice and an even greater decrement in *Wwtr1*^Oc-cKO^ null mice, indicating a gene-dosage effect of Wwtr1 on osteoblast-mediated bone formation. **e** TRAP staining (red color) for osteoclast activity. Data are expressed as the mean ± S.D. from serum samples of individual mice (*n* = 6). *P < 0.05, **P < 0.01, ***P < 0.001 compared with *wild-type* control mice. *P* values were determined by 1-way ANOVA with Tukey’s multiple-comparisons test

In the osteoblast specific conditional *Wwtr1* knockout mouse model, we found a gene-dosage effect on osteoblast-mediated bone formation and bone mass (Figs. 1 and S1). Compared with control mice (*Wwtr1*^+/+^), heterozygous conditional *Oc*-Cre;*Wwtr1*^flox/+^ (*Wwtr1*^Oc-Het^), which has an approximately 37% reduction in *Wwtr1* message expression in bone (Table 1), showed 15% and 12% reductions of BMD in male and female adult mice, respectively (Fig. 1a, b). *Oc*-Cre;*Wwtr1*^flox/−^ (*Wwtr1*^Oc-cKO^) mice, which had a 64% reduction in *Wwtr1* message expression in bone, showed an even greater loss of bone mass, with 24% and 22% reductions of BMD in male and female adult mice, respectively. Micro-CT 3D analysis showed that the reduction in bone mass in single conditional *Wwtr1*^Oc-Het^ heterozygous mice arose from a 23% reduction in trabecular bone volume and a 11% reduction in cortical bone thickness in both male and female adult mice. Conditional *Wwtr1*^Oc-cKO^ mice had a 44% loss in trabecular volume and 20% loss in cortical thickness.

**Table 1:** Gene-expression profiles in bone from *Wwtr1-*deficient mice

Gene	*Wild-type*	*Wwtr1*^*+/−*^	*Wwtr1*^*Oc-Het*^	*Wwtr1*^*Oc-cKO*^	*P*-value
**Osteoblast lineage**					
*Pkd1*	1.00 ± 0.15	1.03 ± 0.22	0.99 ± 0.34	0.96 ± 0.32	0.971 8
*Wwtr1*	1.00 ± 0.24	0.61 ± 0.10***	0.63 ± 0.14***	0.36 ± 0.09***	<0.000 1
*Fgf23*	1.00 ± 0.10	0.97 ± 0.22	1.61 ± 0.45*	2.31 ± 0.61***	<0.000 1
*Runx2-II*	1.00 ± 0.13	0.80 ± 0.21	0.82 ± 0.27	0.61 ± 0.10**	0.017 7
*Osteopontin*	1.00 ± 0.26	0.83 ± 0.20	0.67 ± 0.11*	0.62 ± 0.21*	0.014 3
*Bsp*	1.00 ± 0.11	0.94 ± 0.28	0.68 ± 0.16*	0.54 ± 0.15***	0.000 3
*Mepe*	1.00 ± 0.12	1.04 ± 0.22	1.43 ± 0.28**	1.81 ± 0.22***	<0.000 1
*Col1*	1.00 ± 0.20	0.81 ± 0.23	0.67 ± 0.21*	0.65 ± 0.18*	0.030 4
*Alp*	1.00 ± 0.20	1.01 ± 0.29	0.69 ± 0.11*	0.52 ± 0.15***	0.000 2
*Osteocalcin*	1.00 ± 0.19	0.80 ± 0.23	0.53 ± 0.19**	0.57 ± 0.15**	0.001 3
*OPG*	1.00 ± 0.13	0.97 ± 0.29	0.89 ± 0.31	0.96 ± 0.24	0.892 6
*RankL*	1.00 ± 0.17	1.13 ± 0.24	1.12 ± 0.11	1.52 ± 0.15***	<0.000 1
*Fzd2*	1.00 ± 0.25	1.02 ± 0.18	1.03 ± 0.18	0.96 ± 0.28	0.935 4
*Wnt10b*	1.00 ± 0.08	1.08 ± 0.28	0.97 ± 0.17	0.73 ± 0.06*	0.016 4
*CYR61*	1.00 ± 0.13	1.06 ± 0.10	1.54 ± 0.25**	2.06 ± 0.55***	<0.000 1
*CTGF*	1.00 ± 0.10	1.06 ± 0.28	1.18 ± 0.41	1.54 ± 0.31*	0.022 1
**Osteoclast**					
*Trap*	1.00 ± 0.15	0.95 ± 0.19	1.10 ± 0.22	1.41 ± 0.19**	0.002 1
*Mmp9*	1.00 ± 0.14	0.85 ± 0.22	0.87 ± 0.15	0.86 ± 0.18	0.380 8
**Chondrocyte**					
*Collagen II*	1.00 ± 0.20	0.86 ± 0.20	0.79 ± 0.26	0.67 ± 0.15*	0.045 8
*Collagen X*	1.00 ± 0.21	0.87 ± 0.36	0.74 ± 0.39	0.45 ± 0.23*	0.033 9
**Adipocyte**					
*PPARγ2*	1.00 ± 0.17	1.23 ± 0.14	1.57 ± 0.30**	2.03 ± 0.42***	<0.000 1
*aP2*	1.00 ± 0.18	1.27 ± 0.31	1.64 ± 0.29**	2.51 ± 0.36***	<0.000 1
*Lpl*	1.00 ± 0.15	1.31 ± 0.37	1.63 ± 0.34*	2.56 ± 0.47***	<0.000 1

Data are mean ± S.D. from 6 tibias of *wild-type* control, *Wwtr1*^+/−^, *Wwtr1*^Oc-Het^, and *Wwtr1*^Oc-cKO^ mice and expressed as the fold changes relative to the housekeeping gene *β-actin* subsequently normalized to control mice. *, **, *** indicates significant difference from *wild-type* control at *P* < 0.05, *P* < 0.01, *P* < 0.001, respectively. *P* values were determined by 1-way ANOVA with Tukey’s multiple-comparisons test

In agreement with the DEXA and micro-CT data, analysis of bone histology showed a *Wwtr1* gene-dosage dependent reduction in trabecular bone volume (Figs. 1c and S1a) and cortical bone thickness (Figs. 1c and S1b) in the distal femur and a decrease in bone formation rate measured by double calcein labeling (Fig. 1d). There were 43% and 55% reductions of periosteal MAR in both conditional *Wwtr1*^Oc-Het^ heterozygous and *Wwtr1*^Oc-cKO^ null mice, respectively, compared with control mice (*Wwtr1*^+/+^). Unexpectedly, conditional deletion of *Wwtr1* in osteoblasts resulted in enhanced osteoclast activity, as evidenced by increased TRAP immunostaining in the growth plate of conditional *Wwtr1*^Oc-cKO^ null mice (Fig. 1e).

Real-time RT-PCR analysis revealed a *Wwtr1* gene-dosage dependent changes in osteoblast markers including *Runx2*-II and *Wnt10b, FGF-23*, *Mepe*, *RankL*, *CYR61*, and *CTGF* and chondrocyte markers such as *Collagen II* and *Collagen X*. Reductions of *Runx2-II* and *Wnt10b* impairs osteoblast proliferation and differentiation. Increments of *FGF-23* and *Mepe* as well as Yap signaling such as increased *CYR61* and *CTGF* gene expressions inhibits osteoblast differentiation and mineralization. An increase in *RankL* and the *RankL*/OPG ratio promotes osteoclast differentiation, leading to greater TRAP staining and higher osteoclast activity in conditional *Wwtr1*^Oc-cKO^ null mice compared with control mice (*Wwtr1*^+/+^). Conditional deletion of *Wwtr1* also resulted in increased adipocyte markers such as *PPARγ2*, *aP2* and *Lpl* gene expressions (Table 1).

Unexpectedly, global *Wwtr1*^+/−^ heterozygous mice, which had a ~40% reduction in *Wwtr1* message expression in bone, did not have significant changes in BMD or bone volume. Single-heterozygous *Wwtr1*^+/–^ showed normal bone gene expression profiles as well (Table 1). The osteoblast specific reduction but not global reductions of *Wwtr1* on the skeletal phenotype points to other important co-factors in osteoblasts, such as Wwtr1 interactions with PC1, or counteracting effects of Wwtr1 in non-osteoblastic cells.

### Additive effects of combined *Pkd1* and *Wwtr1* deficiency to reduce bone mass

**Fig. 2:**
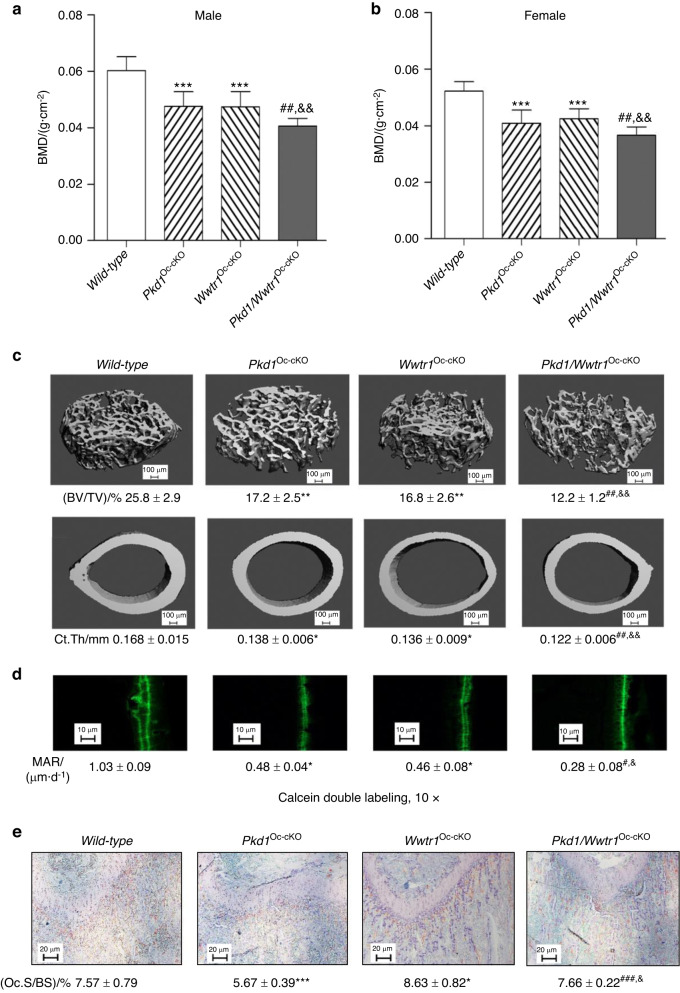
Conditional deletion of *Pkd1* and *Wwtr1* in mature osteoblasts on postnatal bone homeostasis. **a**, **b** Bone mineral density by DEXA scan in male and female mice. **c** Bone structure by micro-CT 3D images analysis from male mice. **d** Periosteal mineral apposition rate (MAR) by Calcein double labeling. There was a significant reduction in periosteal MAR in single *Pkd1*^Oc-cKO^ or *TAZ*^Oc-cKO^ mice compared with age-matched control mice and an even greater decrement in double *Pkd1/TAZ*^*Oc-cKO*^ null mice, indicating an additive effect of PC1 and TAZ on osteoblast-mediated bone formation. **e** TRAP staining (red color) for osteoclast activity. Data are expressed as the mean ± S.D. from serum samples of individual mice (*n* = 6). *P < 0.05, **P < 0.01, ***P < 0.001 compared with *wild-type* mice, ^#^*P* < 0.05, ^##^*P* < 0.01, ^###^*P* < 0.001 compared with *Wwtr1*^*Oc-cKO*^ mice, and ^&^*P* < 0.05, ^&&^*P* < 0.01, ^&&&^*P* < 0.001 compared with *Pkd1*^*Oc-cKO*^ mice, respectively. *P* values were determined by 1-way ANOVA with Tukey’s multiple-comparisons test

To test the functional effects of the interaction between Pkd1 and Wwtr1 in osteoblasts, we characterized *Osteocalcin*-Cre;*Pkd1*^flox/−^; *Wwtr1*^flox/−^ (*Pkd1*/*Wwtr1*^Oc-cKO^) double null mice with osteoblast-specific conditional deletions of both *Pkd1 and Wwtr1* in bone. In the conditional *Pkd1*/ *Wwtr1* double knockout model, we found that *Pkd1* and *Wwtr1* have additive effects in reducing osteoblast-mediated bone formation. Compared with control mice (*Pkd1*^flox/+^*;Wwtr1*^flox/+^), both conditional *Pkd1*^Oc-cKO^ and *Wwtr1*^Oc-cKO^ null mice had similar reductions in bone mass, as evidenced by respective 22% and 21% reductions of BMD (Fig. 2a, b), 33% and 35% reductions in trabecular bone volume (Figs. 2c and S2a), and 18% and 19% reductions in cortical bone thickness (Figs. 2c and S2b) as determined by distal femur Micro-CT 3D image analysis and Goldner staining. Also, the reductions in bone mass were similar in male and female adult mice (Fig. 2a, b).

The skeletal phenotype of double *Pkd1*/*Wwtr1*^Oc-cKO^ mice was more severe than either single *Pkd1*^Oc-cKO^ or *Wwtr1*^Oc-cKO^ null mice. Double *Pkd1*/*Wwtr1*^Oc-cKO^ mice had greater losses in BMD, trabecular bone volume, and cortical bone thickness with 33%, 53%, and 27% reductions, respectively in both male and female adult mice. This indicates the additive effects of Pkd1 and Wwtr1 in postnatal bone homeostasis (Figs. 2 and S2). Consistent with lower bone mass, combined *Pkd1* and *Wwtr1* deficiency also resulted in additive reductions in osteoblast-related gene expressions, such as in *Runx2-II*, *Osteocalcin*, and *Dmp1* (Table 2), as well as mechanosensing responsive genes such as in *Wnt10b, c-Jun, and PTGS2* (Table 2). Periosteal MAR (Fig. 2d) was decreased by ~73% in conditional double *Pkd1/Wwtr1*^*Oc-cKO*^ mice compared to controls, whereas *Wwtr1*^*Oc-cKO*^ and *Pkd1*^*Oc-cKO*^ single conditional knockout mice had reductions in periosteal MAR of 55% and 53%, respectively compared to control mice. Loss of either *Pkd1* or *Wwtr1* resulted in enhanced marrow adipogenesis, but no additive effects on adipocyte differentiation-related gene expressions were observed in the conditional double *Pkd1/Wwtr1*^*Oc-cKO*^ mice (Table 2).

**Table 2:** Gene-expression profiles in bone from *Pkd1*^Oc-cKO^ or/and *Wwtr1*^Oc-cKO^ mice

Gene	*Wild-type*	*Pkd1*^Oc-cKO^	*Wwtr1*^Oc-cKO^	*Pkd1/Wwtr1*^*Oc-cKO*^	*P*-value
**Osteoblast lineage**					
*Pkd1*	1.00 ± 0.11	0.41 ± 0.10***	0.93 ± 0.31	0.45 ± 0.10^&&&^	<0.000 1
*Wwtr1*	1.00 ± 0.24	0.98 ± 0.27	0.46 ± 0.10***	0.43 ± 0.15^###^	<0.000 1
*Pkd2*	1.00 ± 0.12	1.05 ± 0.21	0.93 ± 0.25	1.09 ± 0.31	0.627 1
*Runx2-II*	1.00 ± 0.14	0.76 ± 0.11**	0.75 ± 0.10*	0.62 ± 0.21^#,&^	0.001 7
*Osteocalcin*	1.00 ± 0.13	0.75 ± 0.15**	0.78 ± 0.11**	0.66 ± 0.11^#,&^	0.001 0
*FGF-23*	1.00 ± 0.13	0.55 ± 0.17*	2.30 ± 0.56***	1.02 ± 0.25	<0.000 1
*Dmp1*	1.00 ± 0.20	0.80 ± 0.10*	0.78 ± 0.14*	0.65 ± 0.10^#,&^	0.001 2
*Wnt10b*	1.00 ± 0.04	0.77 ± 0.20**	0.73 ± 0.19**	0.63 ± 0.11^#,&^	0.001 4
*Wnt1*	1.00 ± 0.21	0.70 ± 0.16*	0.92 ± 0.35	0.52 ± 0.21^#,&&&^	0.000 7
*FzD2*	1.00 ± 0.33	0.92 ± 0.17	0.93 ± 0.24	0.55 ± 0.19^#,&^	0.001 6
*c-Jun*	1.00 ± 0.24	0.95 ± 0.28	1.07 ± 0.22	0.62 ± 0.12^#,&&^	0.000 9
*c-Fos*	1.00 ± 0.20	0.92 ± 0.21	0.74 ± 0.15*	0.67 ± 0.13^###^	0.001 1
*Axin-2*	1.00 ± 0.31	0.77 ± 0.10*	0.88 ± 0.20	0.67 ± 0.13^&^	0.001 2
*PTGS2*	1.00 ± 0.27	0.70 ± 0.15*	0.68 ± 0.16**	0.44 ± 0.19^#,&^	<0.000 1
*OPG*	1.00 ± 0.19	0.93 ± 0.26	1.08 ± 0.26	0.97 ± 0.18	0.708 0
*RankL*	1.00 ± 0.15	0.73 ± 0.14*	1.45 ± 0.13***	0.89 ± 0.18^#,&&&^	<0.000 1
*CYR61*	1.00 ± 0.13	1.40 ± 0.18*	1.94 ± 0.51***	1.67 ± 0.33	0.000 5
*CTGF*	1.00 ± 0.10	1.38 ± 0.23*	1.68 ± 0.30**	1.55 ± 0.36	0.001 4
**Osteoclast**					
*Trap*	1.00 ± 0.15	0.63 ± 0.14**	1.43 ± 0.20***	0.93 ± 0.14^##,&&&^	<0.000 1
*Mmp9*	1.00 ± 0.14	0.63 ± 0.08*	0.86 ± 0.18	0.95 ± 0.35^#^	0.031 7
**Chondrocyte**					
*Collagen II*	1.00 ± 0.19	0.92 ± 0.24	0.73 ± 0.15*	0.51 ± 0.07^##,&^	<0.000 1
*VegfA*	1.00 ± 0.38	1.74 ± 0.22*	1.66 ± 0.49*	1.70 ± 0.57	0.014 3
**Adipocyte**					
*PPARγ2*	1.00 ± 0.20	1.78 ± 0.34**	1.88 ± 0.51**	1.61 ± 0.22	0.001 0
*aP2*	1.00 ± 0.16	1.71 ± 0.33***	2.09 ± 0.31***	1.77 ± 0.38	<0.000 1
*Lpl*	1.00 ± 0.13	1.62 ± 0.28**	2.01 ± 0.51***	1.88 ± 0.38	0.000 4

Data are mean ± S.D. from 6 tibias of *wild-type* control, *Pkd1*^Oc-cKO^, *Wwtr1*^Oc-cKO^, and *Pkd1*/*Wwtr1*^*Oc-*cKO^ mice and expressed as the fold changes relative to the housekeeping gene *β-actin* subsequently normalized to control mice. *P < 0.05, **P < 0.01, ***P < 0.001 compared with *wild-type* mice, ^#^*P* < 0.05, ^##^*P* < 0.01, ^###^*P* < 0.001 compared with *Wwtr1*^*Oc-cKO*^ mice, and ^&^*P* < 0.05, ^&&^*P* < 0.01, ^&&&^*P* < 0.001 compared with *Pkd1*^*Oc-cKO*^ mice, respectively. *P* values were determined by 1-way ANOVA with Tukey’s multiple-comparisons test

We found that the conditional deletion of *Pkd1* or *Wwtr1* in osteoblasts has opposite effects on osteoclast activities. There was a decreased *RankL/OPG* expression ratio and TRAP immunostaining in *Pkd1*^*Oc-cKO*^ mice but an increased *RankL/OPG* expression ratio and TRAP immunostaining in *Wwtr1*^*Oc-cKO*^ mice (Table 2 and Fig. 2e). In contrast, double *Pkd1/Wwtr1*^*Oc-cKO*^ had similar *RankL* expression and TRAP immunostaining when compared to control, indicating a recovery of osteoclast activities in the double null mice (Table 2 and Fig. 2e). Changes in gene expression and immunostaining in bone correlated with alterations in serum biomarkers (Table 3). In this regard, further evidence for osteoblast and osteocyte dysfunction includes reductions in serum osteocalcin and FGF-23 from in single *Pkd1*^Oc-cKO^ or *Wwtr1*^Oc-cKO^ mice compared with age-matched control mice and an even greater decrement in double *Pkd1/Wwtr1*^*Oc-cKO*^ null mice (Table 3). In contrast, serum levels of TRAP, a marker of bone resorption, were decreased in single *Pkd1*^Oc-cKO^ mice, increased in single *Wwtr1*^Oc-cKO^ mice, but restored in double *Pkd1/Wwtr1*^*Oc-cKO*^ null mice compared with control littermates (Table 3). In addition, serum level of leptin was significantly higher in single *Pkd1*^Oc-cKO^ or *Wwtr1*^Oc-cKO^ mice than age-matched control mice. However, we did not observe further increase in double *Pkd1/Wwtr1*^*Oc-cKO*^ null mice (Table 3). These findings suggest that Pkd1 and Wwtr1 have distinct functions among osteoblasts, adipocytes, and osteoclasts in bone in vivo.

**Table 3:** Biochemistry analysis of serum from *Pkd1*^Oc-cKO^ or/and *Wwtr1*^Oc-cKO^ mice

Genotype	*Wild-type*	*Pkd1*^Oc-cKO^	*Wwtr1*^Oc-cKO^	*Pkd1/Wwtr1*^Oc-cKO^	*P*-value
Urea nitrogen/(mg·dL^−1^)	19 ± 5	22 ± 5	21 ± 6	22 ± 4	0.723 6
Calcium/(mg·dL^−1^)	10.3 ± 0.9	10.4 ± 1.3	10.2 ± 1.5	9.4 ± 0.9	0.399 0
Phosphorus/(mg·dL^−1^)	10.1 ± 1.9	10.4 ± 1.4	9.8 ± 2.4	10.3 ± 1.5	0.935 5
Osteocalcin (ng/mL)	17.5 ± 3.5	3.4 ± 0.7***	10.7 ± 2.6***	1.0 ± 0.8^#,&&&^	<0.000 1
TRAP/(U·L^−1^)	10.8 ± 1.3	7.6 ± 0.6***	12.2 ± 1.9*	9.8 ± 1.1^##,&&^	<0.000 1
FGF23/(pg·mL^−1^)	117 ± 32	79 ± 16*	116 ± 35	46 ± 16^#,&&&^	0.070 4
Leptin/(pg·mL^−1^)	836 ± 200	2 636 ± 759***	2 649 ± 915***	1 793 ± 600^###,&&^	<0.000 1

Data are mean ± S.D. from 6 serum samples of *wild-type* control, *Pkd1*^Oc-cKO^, *Wwtr1*^Oc-cKO^, and *Pkd1*/*Wwtr1*^*Oc-*cKO^ mice. *P < 0.05, **P < 0.01, ***P < 0.001 compared with *wild-type* mice, ^#^*P* < 0.05, ^##^*P* < 0.01, ^###^*P* < 0.001 compared with *Wwtr1*^*Oc-cKO*^ mice, and ^&^*P* < 0.05, ^&&^*P* < 0.01, ^&&&^*P* < 0.001 compared with *Pkd1*^*Oc-cKO*^ mice, respectively. *P* values were determined by 1-way ANOVA with Tukey’s multiple-comparisons test

### Loss of mechanical loading response in conditional *Pkd1* and *Wwtr1* deficient mice

**Fig. 3:**
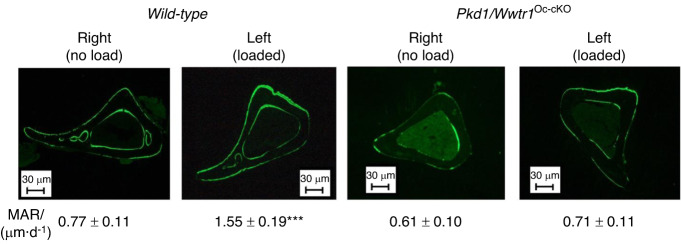
An impairment of anabolic response to mechanical loading in conditional *Pkd1* and *Wwtr1* deletion in bone. Representative images of midshaft tibia cross sections from no-load and loaded ulnae of *wild-type* control and compound *Pkd1/Wwtr1*^*Oc-cKO*^ null mice after loading. Data are mean ± S.D. from 6 tibias of *wild-type* control and *Pkd1/Wwtr1*^*Oc-cKO*^ mice. ***P < 0.001 compared with *wild-type* mice. *P* values were determined by 1-way ANOVA with Tukey’s multiple-comparisons test

The cross-sections of tibiae from control and double *Pkd1/Wwtr1*^*Oc-cKO*^ null mice after mechanical tibia loading studies in vivo are shown in Fig. 3. In *wild-type* control mice, loaded tibia showed a 2-fold increase in periosteal mineral apposition rate. In contrast, there was no measurable increase in periosteal mineral apposition in the loaded tibia from double *Pkd1*/*Wwtr1* knockout mice (Fig. 3). In addition, a real-time RT-PCR analysis revealed that loaded tibia from the control mice had a dramatic response to mechanical stimulation, evidenced by significant increases of mechanosensing (e.g., *Wnt10b*, *FzD2*, *Axin2*, *PTGS2*, *c-Jun*, *c-Fos*, and *Runx2*-II) and osteogenic (e.g., *Osteocalcin*, *Alp*, *Collagen I*, and *Dmp1*) but decreases of adipogenic (e.g., *PPARγ2*, *aP2*, and *Lpl*) gene expressions when compared with no load control tibia. In contrast, even when using the same loading regimen, no changes of mechanosensing, osteogenic, and adipogenic gene expression profiles were observed in the loaded tibia from double *Pkd1/ Wwtr1*^*Oc-cKO*^ null mice when compared with no load control tibia (Table 4). Thus, PC1 and Wwtr1 are important in mediating mechanotransduction in bone.

**Table 4:** Mechanosensing, osteogenic, and adipogenic gene-expression profiles that respond to mechanical loading in *wild-type* and double *Pkd1*/*Wwtr1*^Oc-cKO^ mice

Gene	*Wild-type* (no load)	*Wild-type* (loaded)	*Pkd1/Wwtr1*^Oc-cKO^ (no load)	*Pkd1/Wwtr1*^Oc-cKO^ (loaded)
*Wnt10b*	1.00 ± 0.32	1.74 ± 0.62**	1.00 ± 0.31	0.94 ± 0.31
*c-Jun*	1.00 ± 0.27	1.72 ± 0.41***	1.00 ± 0.18	0.95 ± 0.29
*c-Fos*	1.00 ± 0.22	2.04 ± 0.66***	1.00 ± 0.24	0.92 ± 0.29
*Axin-2*	1.00 ± 0.16	1.54 ± 0.25***	1.00 ± 0.19	0.92 ± 0.33
*PTGS2*	1.00 ± 0.16	3.34 ± 1.05***	1.00 ± 0.22	1.15 ± 0.17
*Runx2-II*	1.00 ± 0.28	1.94 ± 0.54***	1.00 ± 0.19	0.92 ± 0.19
*FzD2*	1.00 ± 0.26	1.92 ± 0.47***	1.00 ± 0.26	0.93 ± 0.32
*Osteocalcin*	1.00 ± 0.34	5.26 ± 1.58***	1.00 ± 0.36	1.77 ± 0.41
*Alp*	1.00 ± 0.32	2.82 ± 1.05***	1.00 ± 0.30	0.92 ± 0.29
*Collagen I*	1.00 ± 0.25	4.93 ± 1.59***	1.00 ± 0.58	1.01 ± 0.33
*Dmp1*	1.00 ± 0.32	1.83 ± 0.29***	1.00 ± 0.34	1.03 ± 0.27
*PPARγ2*	1.00 ± 0.40	0.24 ± 0.12***	1.00 ± 0.21	0.86 ± 0.28
*aP2*	1.00 ± 0.25	0.57 ± 0.14***	1.00 ± 0.18	0.89 ± 0.17
*Lpl*	1.00 ± 0.32	0.35 ± 0.11***	1.00 ± 0.30	0.88 ± 0.30

Data are mean ± S.D. from 6 tibiae of *wild-type* control and *Pkd1*/*Wwtr1*^*Oc-*cKO^ mice and expressed as the fold changes relative to the housekeeping gene *eEF1a1* subsequently normalized to control or *Pkd1*/*Wwtr1*^Oc-cKO^ no load tibiae. *, **, *** indicates significant difference from *wild-type* or *Pkd1*/*Wwtr1*^Oc-cKO^ no load control at *P* < 0.05, *P* < 0.01, *P* < 0.001

### Validation of MS2 key binding to residues in PC1/PC2 C-terminus tails

**Fig. 4:**
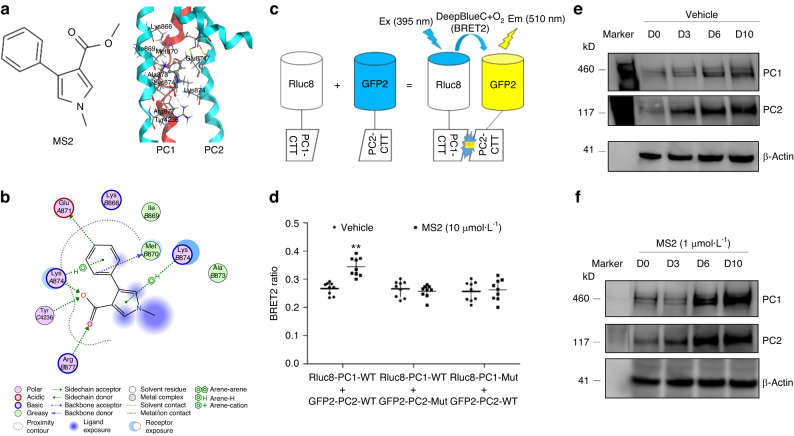
Cell-based BRET^2^ assays for MS2-target engagement assays. **a** Chemical structure of MS2 and an example of predicted 3D binding mode for MS2 (ball-and-sticks rendering in CPK colors) in PC2 (light blue). PC1 (red) as bound to PC2 in the homology structure is superimposed. **b** An example of calculated 2D binding mode and residues for MS2 in PC1/PC2 C-tails. **c** A diagram of BRET^2^ constructs and reactions in the presence of DeepBlue C with or without MS2 stimulation. **d** BRET^2^ signal changes from *wild-type* and mutant constructs with or without MS2 incubation. **e**, **f** Time-dependent changes of PC1 and PC2 proteins with or without MS2 incubation during osteogenic differentiation cultures in MC3T3-E1 cell line. Incubation Data are presented as the mean ± SD from 3 independent experiments (*n* = 3). **P < 0.01 compared with vehicle control

We have previously showed that the small molecule MS2 activates PC1/PC2 complex signaling.^2^ Using computational modeling, we engaged in an induced fit docking campaign and predicted several potential ligand binding complexes. From these predicted poses, we identified key residues in the PC1 and PC2 C-terminus tail regions with which MS2 is predicted to interact. As shown in Fig. 4, the PC2-CTT binding site for MS2 is predicted to include Lysine^874^ and Arginine^877^, whereas the PC1-CTT binding site for MS2 involves Tyrosine^4236^ (Fig. 4a, b). To test these predictions, we performed site-mutagenesis of key residues in both PC1 and PC2 and tested the effects of MS2 on PC1 and PC2 assembly using a BRET^2^ assay. We observed that the compound MS2 markedly enhances the BRET^2^ signal in *wild-type* constructs, while mutagenesis of key residues in either PC1-CTT or PC2-CTT constructs completely abolished the BRET^2^ signal in the presence of compound MS2, confirming the role of MS2 in enhancing PC1 and PC2 interactions and lending support that MS2 is binding to the protein complex (Fig. 4c, d).

Next, we examined PC1/PC2 complex formation during MC3T3-E1 osteoblast differentiation in vitro. We observed culture duration dependent increase of PC1/PC2 complex formation by western blot analysis. Incubation with 1 μmol·L^−1^ of MS2 in osteogenic cultures markedly increased the amount of PC1 and PC2 protein as assessed by western blot analysis (Fig. 4e, f). These data suggests that MS2 may molecularly stabilize the PC1/PC2 complex in osteoblast culture in vitro.

### Loss of MS2-mediated stimulated increase in bone mass in conditional *Pkd1* and *Wwtr1* deficiency mice

**Fig. 5:**
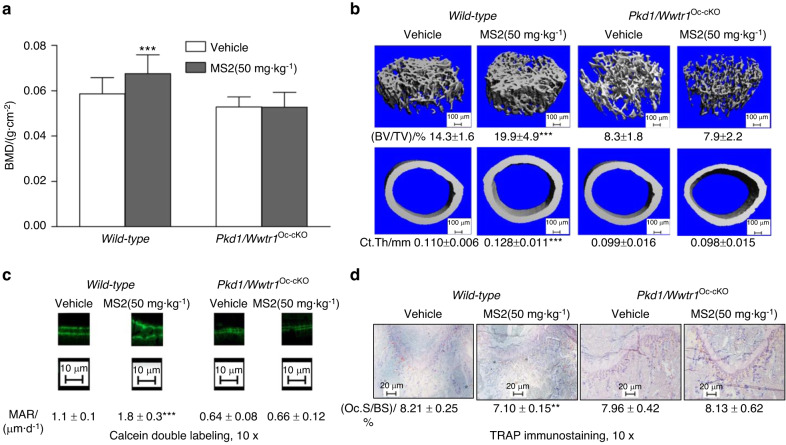
Effects of MS2 on bone formation in *wild-type* and compound *Pkd1/Wwtr1*^*Oc-cKO*^ null mice. **a** Bone mineral density by DEXA scan. **b** Bone structure by micro-CT 3D images analysis. **c** Periosteal mineral apposition rate (MAR) by Calcein double labeling. **d** Osteoclast activities by TRAP immunostaining after MS2 (50 mg·kg^−1^) treatment for 4 weeks compared to vehicle control. Data are mean ± S.D. from 6 tibias of *wild-type* control and compound *Pkd1/Wwtr1*^Oc-cKO^ null mice. *P < 0.05, **P < 0.01, ***P < 0.001 compared with *wild-type* control mice. *P* values were determined by 1-way ANOVA with Tukey’s multiple-comparisons test

Finally, we treated *wild-type* and conditional double *Pkd1/Wwtr1*^*Oc-cKO*^ null mice with vehicle or MS2 (50 mg·kg^−1^) i.p. daily and assessed their skeletal response. After only 2 weeks, we observed that *wild-type* control mice treated with MS2 had a 15% increment in femoral bone mineral density compared to vehicle control (Fig. 5). Micro-CT 3D images revealed that MS2 treated *wild-type* mice had a 39% increase in trabecular bone volume and 16% increase in cortical bone thickness.

In contrast, administration of MS2 had no effects on bone mineral density and bone structure in double *Pkd1/Wwtr1*^Oc-cKO^ null mice, suggesting specific-target dependent effects of MS2 on polycystins/Wwtr1 signaling (Fig. 5). We also observed that there were 1.6-fold increases in bone formation rate in *wild-type* mice treated with MS2 compared to the vehicle control, in agreement with enhanced osteoblastogenesis (e.g., *Runx2-II*, *Osteocalcin, ALP* and *Dmp1*) and suppressed marrow adipogenesis (e.g., *PPARγ2*, *aP2*, and *Lpl*) by a real-time RT-PCR analysis (Table 5 and Fig. 5). Again, administration of MS2 had no effects on bone formation rate and bone gene expression profiles in double *Pkd1/Wwtr1*^Oc-cKO^ null mice. Furthermore, MS2 stimulated mechanosensing gene expressions, including *Wnt1*, *Wnt10b*, *Axin-2*, *FzD2*, *c-Jun*, *c-Fos*, *eNOS*, and *PTGS2*, consistent with MS2 acting as a small molecule “mechanomimetic”. MS2 treatment decreased *RankL*/*OPG* expression ratio and TRAP immunostained osteoclasts in the MS2-treated mice compared to vehicle control (Table 5 and Fig. 5). Administration of MS2 had no effects on osteoblast-mediated bone formation rate, marrow adipogenesis, and osteoclast activity in conditional double *Pkd1/Wwtr1*^*Oc-cKO*^ null mice (Table 5 and Fig. 5). These data support that MS2 functions as anabolic drugs through the polycystins/Wwtr1 pathway to promote the bone remodeling process.

**Table 5:** Gene expression profiles in bone from MS2-treated *wild-type* control and *Pkd1*/*Wwtr1*^Oc-cKO^ mice

Gene	*Wild-type* (Vehicle)	*Wild-type* (MS2)	*Pkd1/Wwtr1*^*Oc-cKO*^ (Vehicle)	*Pkd1/Wwtr1*^*Oc-cKO*^ (MS2)
*Pkd1*	1.00 ± 0.13	0.94 ± 0.15	1.00 ± 0.37	1.03 ± 0.28
*Wwtr1*	1.00 ± 0.32	1.01 ± 0.32	1.00 ± 0.40	0.82 ± 0.19
*Runx2-II*	1.00 ± 0.19	1.79 ± 0.52**	1.00 ± 0.32	1.03 ± 0.28
*Osteocalcin*	1.00 ± 0.24	1.68 ± 0.24***	1.00 ± 0.57	0.86 ± 0.61
*ALP*	1.00 ± 0.14	1.56 ± 0.23***	1.00 ± 0.52	1.09 ± 0.55
*Dmp1*	1.00 ± 0.18	1.45 ± 0.17***	1.00 ± 0.33	1.02 ± 0.48
*OPG*	1.00 ± 0.38	1.10 ± 0.37	1.00 ± 0.30	1.18 ± 0.33
*RankL*	1.00 ± 0.30	0.57 ± 0.15*	1.00 ± 0.50	0.99 ± 0.33
*Trap*	1.00 ± 0.26	0.56 ± 0.10*	1.00 ± 0.58	0.93 ± 0.28
*PPARγ2*	1.00 ± 0.43	0.59 ± 0.11*	1.00 ± 0.47	1.06 ± 0.18
*aP2*	1.00 ± 0.22	0.60 ± 0.26*	1.00 ± 0.22	1.07 ± 0.12
*Lpl*	1.00 ± 0.28	0.63 ± 0.12*	1.00 ± 0.40	0.95 ± 0.39
*Wnt1*	1.00 ± 0.30	3.02 ± 0.98***	1.00 ± 0.60	0.97 ± 0.40
*Wnt10b*	1.00 ± 0.15	1.52 ± 0.25***	1.00 ± 0.40	1.12 ± 0.21
*FzD2*	1.00 ± 0.26	3.36 ± 1.56***	1.00 ± 0.30	0.95 ± 0.18
*c-Fos*	1.00 ± 0.20	1.47 ± 0.34*	1.00 ± 0.22	1.00 ± 0.27
*c-Jun*	1.00 ± 0.23	1.74 ± 0.24***	1.00 ± 0.29	0.84 ± 0.20
*Axin-2*	1.00 ± 0.22	2.57 ± 1.02**	1.00 ± 0.16	0.92 ± 0.17
*PTGS2*	1.00 ± 0.35	2.25 ± 0.78**	1.00 ± 0.26	0.90 ± 0.22
*eNOS*	1.00 ± 0.45	3.09 ± 0.68***	1.00 ± 0.13	1.03 ± 0.22

Data are mean ± S.D. from 6 tibias of *wild-type* control and *Pkd1*/*Wwtr1*^Oc-cKO^ mice and expressed as the fold changes relative to the housekeeping gene *eEF1a1* subsequently normalized to control or *Pkd1*/*Wwtr1*^Oc-cKO^ no load tibias. *, **, *** indicates significant difference from *wild-type* control at *P* < 0.05, *P* < 0.01, *P* < 0.001

## Discussion

In the current study, we found that conditional deletion of both *Pkd1* and *Wwtr1* in osteoblasts using *Oc*-Cre in double *Pkd1/Wwtr1*^*Oc-cKO*^ null mice resulted in a more severe skeletal phenotype than loss of either PC1 or WWTR1 alone in the single *Pkd1*^*Oc-cKO*^ or *Wwtr1*^*Oc-cKO*^ null mice. Indeed, double *Pkd1*/*Wwtr1*^Oc-cKO^ null mice had greater reductions of bone mineral density and periosteal mineral apposition rate compared to single *Wwtr1*^*Oc-cKO*^ or *Pkd1*^*Oc-cKO*^ null mice. Micro-CT 3D image analysis revealed that this was due to greater loss in both trabecular bone volume and cortical bone thickness in double *Pkd1*/ *Wwtr1*^Oc-cKO^ null mice compared to single *Pkd1*^Oc-cKO^ or *Wwtr1*^Oc-cKO^ null mice. Double *Pkd1*/ *Wwtr1*^Oc-cKO^ null mice also exhibited greater reductions in osteoblast-related message expression in bone compared to single *Pkd1*^Oc-cKO^ or *Wwtr1*^Oc-cKO^ null mice. Moreover, double *Pkd1*/*Wwtr1*^Oc-cKO^ null mice in osteoblasts resulted in greater reductions in mechanosensing responsive gene expressions in physiological conditions and failed to respond to mechanical loading in vivo. Control mice treated with the PC1/PC2 agonist MS2 had significant increases in femoral BMD and periosteal MAR, whereas MS2 had no anabolic effects on double *Pkd1*/*Wwtr1*^Oc-cKO^ mice. Thus, both loss-of-function genetic and gain-of-function pharmacological studies support co-dependent functions of PC1 and Wwtr1 in regulating osteoblast-mediated bone formation and post-natal bone homeostasis. These functional interactions are consistent with the fact that Wwtr1 binds to the C-terminal tail of PC1 and translocates to the nucleus in response to PC1/PC2 activation.^2,17^

The PC1/Wwtr1 signaling pathway may impact bone formation and bone mass by several cellular mechanisms. First, both PC1 and Wwtr1 have a direct role in osteoblast function and differentiation. In previous work we and others confirmed that conditional deletion of either *Pkd1* or *Wwtr1* in osteoblasts has a direct role in osteoblast-mediated bone formation.^6,25^ Our current studies extend these findings by demonstrating the additive osteopenia phenotypes in double *Pkd1*/*Wwtr1*^Oc-cKO^ null mice are due to direct loss of both PC1 and/or Wwtr1 signaling in the osteoblast lineage. In addition, control mice treated with MS2 that targets the PC1/PC2/Wwtr1 complex had anabolic effects on osteoblast-mediated bone formation and increased bone mass. The magnitude of the anabolic bone response to MS2 was similar to increments in bone mass in mice treated with PTH analogues and RANKL blocking antibodies that were developed to treat osteoporosis.^26^ However, this response required PC1 and Wwtr1 co-dependent signaling, since the administration of MS2 had no effects on bone formation in double *Pkd1*/*Wwtr1*^Oc-cKO^ null mice. These genetic loss-of-function and pharmacological gain-of-function studies indicate a functional interaction in osteoblast activity between PC1 and Wwtr1 in bone under normal physiological conditions.

Second, PC1 and Wwtr1 function as a mechanosensor in osteoblasts and osteocytes. We reported that conditional deletion of *Pkd1* in osteocytes markedly attenuated mechanical loading-induced bone formation.^3^ The Wwtr1/Yap Hippo pathway is known to play an essential role in mechanosensing of alterations in cell stiffness and extracellular matrix.^13–16^ Recent studies demonstrated that mechanical loading activates the Wwtr1/Yap pathway^27–30^ and enhances Wwtr1/Yap nuclear translocation in response to shear stress in both bone marrow mesenchymal stem cells and MLO-Y4 osteocyte-like cell line.^31,32^ Thus, loss of mechanosensing responses in double *Pkd1*/*Wwtr1*^Oc-cKO^ null mice can be explained by loss of Wwtr1 interactions with PC1 and their co-dependent signaling. Indeed, we confirmed that PC1-CTT interacted with PC2-CTT to produce BRET^2^ signal and that the small molecule MS2 enhanced this interaction and stimulated mechanosensing gene expressions like mechanical loading in *wild-type* control mice acting as a “mechanomimetic”. Prior in vitro studies showed that overexpression of either full-length *PC1* or *PC1-CTT* along with full-length *PC2* constructs markedly increased Wwtr1-induced activation of TEAD activity and that the cleavage of the PC1-C-Tail/Wwtr1 complex by mechanical stretch-induced activation of γ-secretase translocates to the nucleus to stimulate Runx2*- and* Wwtr1*-*mediated gene transcription, consistent with our proposition that PC1/Wwtr1 functions as a mechanosening complex in bone.^2,17^

Third, PC1 and Wwtr1 have opposite effects on osteoclast mediated bone resorption. Global *Pkd1* heterozygous mice showed fewer osteoclasts in bone as evidenced by a lower number of TRAP positive osteoclasts,^2,3,6^ consistent with lower TRAP levels in ADPKD patients compared to non-ADPKD control.^10–12,33^ Conditional *Pkd1*^Oc-cKO^ null mice in osteoblast lineages with one global null and one conditional knockout alleles had greater reductions in osteoclast activities,^3,6^ indicating a gene-dosage dependent effect of loss-of-PC1 in osteoblasts impacting osteoclast functions, likely through paracrine effects.^34^ Whereas global *Wwtr1* heterozygous mice exhibited no changes in osteoclast activities compared to *wild-type* control mice,^2^ in our current study conditional *Wwtr1*^oc-cKO^ null mice had higher osteoclast activities in bone as evidenced by increased number of TRAP positive osteoclasts. In contrast, double *Pkd1*/*Wwtr1*^Oc-cKO^ mice had normal osteoclast parameters, consistent with offsetting effects of conditional deletion of *Pkd1* and/or *Wwtr1* in osteoblasts on osteoclasts. These findings agree with previous publications regarding the effects of osteoblast-specific deletion of *Pkd1*^2,3,6–8^ or *Wwtr1*^25,27,35^ on osteoclast activity. Indeed, osteocytes can regulate osteoclast activity through the RANKL/OPG paracrine pathway.^36,37^ PC1 and Wwtr1 deficiency resulted in respective decrease and increase in *RANKL* expression but no difference in *OPG* expression in the *Pkd1*^Oc-cKO^ null and *Wwtr1*^*Oc-cKO*^ null mice, this could account for the differential effects of PC1 and Wwtr1 on osteoclast activity in bone. Moreover, MS2 significantly decreased *RANKL*/*OPG* ratio bone and attenuated osteoclast activity. Our understanding of Wwtr1 regulation of osteoclast function is further supported by the observation by Yang et al. that either global or osteoclast-specific knockout of *Wwtr1* led to a low-bone mass phenotype due to elevated osteoclast formation.^38^ Thus, PC1 and Wwtr1 signaling have divergent effects on osteoclasts and bone resorption.

Finally, PC1 and Wwtr1 regulate adipogenesis in bone marrow. We have previously reported that global PC1 deficiency in mice has an inverse effect, inhibiting osteoblastogenesis and enhancing adipogenesis.^2,7^ Similar to conditional *Pkd1*^Oc-cKO^ null mice,^3,6^ we also observed conditional *Wwtr1*^Oc-cKO^ null mice had greater increments in adipogenic markers than global or conditional *Wwtr1* heterozygous mice in the current study, suggesting a gene-dosage dependent effect of loss-of-Wwtr1 in osteoblasts on bone marrow adipogenesis. Consistent with these loss-of-function results, administration of MS2 or mechanical loading in *wild-type* control mice suppressed marrow adipogenesis but had no effects in double *Pkd1/Wwtr1*^*Oc-cKO*^ null mice. Interestingly, we found a similar increase in adipogenic markers in both PC1 and/or Wwtr1 osteoblast conditional knockout mice, consistent with the phenotypes of senile osteoporosis characterized by decreased osteoblastogenesis and increased adipogenesis leading to increased bone marrow fat.^39–42^ The increase of adipogenic markers could be theoretically explained by increased transdifferentiation of osteoblast precursors to adipocytes,^43,44^ or effects of PC1 and Wwtr1 in osteoblasts/osteocytes differentially releasing paracrine factors that modulate adipogenesis,^45–47^ analogous to paracrine factors that regulate osteoclastogenesis. Regardless, our studies revealed that double *Pkd1*/*Wwtr1*^Oc-cKO^ null mice had no differences in adipogenic markers relative to single *Pkd1*^Oc-cKO^ or *Wwtr1*^Oc-cKO^ null mice, suggesting that PC1 and Wwtr1 regulate adipocyte differentiation through the common polycystins/Wwtr1/PPARγ pathway.^2^

In conclusion, polycystins and Wwtr1 have interdependent effects in mediating bone mechanotransduction, regulating osteoblast mediated bone formation, and the anabolic skeletal responses to a small molecule mechanomimetic and mechanical loading. These observations suggest that the PC1/PC2/ Wwtr1 complex functions through a common pathway and is a novel drug target to treat age-related osteoporosis.^48,49^

## Materials and methods

### Mice

All animal studies were conducted according to guidelines provided by the National Institutes of Health and the Institute of Laboratory Animal Resources, National Research Council. The University of Tennessee Health Science Center’s Animal Care and Use Committee approved all animal studies (Protocol number: 21-0301). We obtained the floxed *Wwtr1* mice (*Wwtr1*^flox/flox^) which harbors two loxP sites flanking exon 2 of the *Wwtr1* gene from Drs. Jeff Wrana and Helen McNeill.^50^ We obtained the floxed *Pkd1* mice (*Pkd1*^flox/flox^) from Dr. Gregory Germino at Johns Hopkins University^51^ and *Oc* (*Osteocalcin*)-Cre transgenic mice from Dr. Thomas Clemens at the University of Alabama.^52^ These mice were bred and maintained on a C57BL/6 J background. At first, we used *Oc*-Cre; *Wwtr1*^+/−^ mice and homozygous *Wwtr1*^flox/flox^ mice to generate conditional *Wwtr1* heterozygous *Oc*-Cre; *Wwtr1*^flox/+^ (*Wwtr1*^Oc-Het^) and homozygous *Oc*-Cre; *Wwtr1*^flox/−^ (*Wwtr1*^Oc-cKO^) null mice as well as *Wwtr1* heterozygous mice (*Wwtr1*^+/−^) and Oc-Cre negative control mice (*Wwtr1*^flox/+^ equivalent to *wild-type*). Second, we also used *Oc*-Cre;*Pkd1*^+/−^ mice and homozygous *Pkd1*^flox/flox^ mice to generate *Oc*-Cre;*Pkd1*^flox/−^ (*Pkd1*^Oc-cKO^) mice. Then we used *Oc*-Cre;*Pkd1*^+/−^;*Wwtr1*^+/−^ mice and homozygous *Pkd1*^flox/flox^;*Wwtr1*^flox/flox^ mice to generate *Oc*-Cre;*Pkd1*^flox/−^;*Wwtr1*^flox/−^ (*Pkd1/Wwtr1*^Oc-cKO^) mice as previously described.^6,9^ These mice were used for skeletal phenotype analysis. The mice were anesthetized with Ketamine (90 mg·kg^−1^) and Xylazine (10 mg·kg^−1^) for LUNAR_PIXIMUS_ bone densitometer scan, and the mice not useful for experimental purposes were sacrificed by CO_2_ inhalation followed by cervical dislocation. In addition, we used wild-type C57BL/6 J mice at 8 weeks of age to examine the effects of MS2 on osteogenesis and adipogenesis in vivo. The mice were treated with intraperitoneal injection of MS2 (50 mg·kg^−1^) i.p. or vehicle control (5% DMSO in PBS solution) once a day for 4 weeks. The bone samples were collected 4 h after the last dose administration.

### Bone densitometry, histomorphometric and micro-CT analysis

BMD of femurs was assessed at 8 weeks of age using a LUNAR_PIXIMUS_ bone densitometer (Lunar Corp, Madison, WI). Calcein (Sigma, St. Louise, MO) double labeling of bone and histomorphometric analyses of periosteal mineral apposition rate (MAR) in tibias and osteoclast surface per bone surface (Oc.S/BS, %) in femurs by TRAP immunostaining were performed using the osteomeasure analysis system (Osteometrics). Bone Masson-Goldner staining was performed by the Histology and histomorphometry Core iLab service from Indiana University School of Medicine. The distal femoral metaphyses were also scanned using a Scanco μCT 40 (Scanco Medical AG, Brüttisellen, Switzerland). A 3D images analysis was done to determine bone volume (BV/TV) and cortical thickness (Ct.Th) as previously described.^3,4,6^

### Real-time quantitative reverse transcription PCR (real-time qRT-PCR) and western blot analysis

For real-time qRT−PCR, 1.0 μg total RNA isolated from either the long bone of 6-week-old mice or 8-days cultured BMSCs in differentiation media was reverse transcribed as previously described.^4,6^ PCR reactions contained 20 ng template (cDNA), 375 nmol·L^−1^ each forward and reverse primers, and 1 X EvaGreen Supermix (Bio-Rad, Hercules, CA) in 10 μL. The threshold cycle (Ct) of tested-gene product from the indicated genotype was normalized to the Ct for cyclophilin A. Then the tested-gene product vs cyclophilin A is normalized to the mean ratio of wild-type or control group, which has been set to 1.

For Western blot analysis, protein concentrations of the supernatant were determined with a total protein assay kit (Bio-Rad, Hercules, CA). Equal quantities of protein were subjected to 4%–12% Bis-Tris or 3%–8% Tris-Acetate gradient Gels (Invitrogen, Carlsbad, CA) and were analyzed with standard western blot protocols as previously described.^4,6^ Polycystin-1 antibody (7E12, sc-130554), Polycystin-1 antibody (C-20, sc-10372), Polycystin-2 antibody (H-280, sc-25749), and Polycystin-2 antibody (YCE2, sc-47734) were purchased from Santa Cruz Biotechnology (Paso Robles, CA). Purified mouse Wwtr1 antibody (560236) was purchased from BD Biosciences (San Jose, CA). Phosphorylated p-Wwtr1 (Ser 89, sc-17610) and β-actin antibody (sc-47778) antibodies were from Santa Cruz Biotechnology (Paso Robles, CA). The intensity of the bands was quantified using Image J software (http://rsb.info.nih.gov/ij/).

### BRET^2^ assays for target engagement assay in vitro

In collaboration with Oak Ridge National Laboratory and The University of Tennessee, Knoxville, we previously identified a compound that is thought to bind to the polycystin1 (PC1-CTT)/polycystin2 (PC2-CTT) complex in their C-terminus tails that we refer to as molecular staple two (MS2).^2^ Here, using computational ligand docking with an initial rigid receptor search using the proxy triangle algorithm and London dG scoring function^53^ and subsequent induced-fit refinement using a “free” receptor geometry, the Amber14SB:EHT force-field,^54,55^ and GBVI/WSA scoring function, as implemented in the Molecular Operating Environment (MOE, 2022.02 Chemical Computing Group ULC, 910–1010 Sherbrooke St. W., Montreal, QC H3A 2R7, Canada, 2023) software package, against our previously identified binding pocket (in a previously published homology model of the PC1/PC2 CTT domain) we predicted over 30 different potential ligand-protein complexes. Investigation of these complexes identified two classes of binding poses, those dominated by PC2-ligand contacts and poses with PC1/PC2 bridging contacts. From the poses containing the largest number of ligand-protein contacts, we identified key residues of MS2 interacting with PC2-CTT [e.g., Arg(R)^877^ and Lys(K)^874^] and PC1-CTT [e.g., Tyr(Y)^4236^]. To confirm the interaction between MS2 and PC1/PC2 C-tail complex (1:3), we used PC1-CTT and PC2 C-terminal tail (PC2-CTT) cDNAs to create Rluc8_PC1-CTT and GFP2_PC2-CTT constructs and develop BRET^2^ assays for their target engagement. The HEK-293T cells were transiently co-transfected with Rluc8_PC1-CTT (3.0 µg) and GFP2_PC2-CTT constructs (3.0 µg) by electroporation using a cell line optimal transfection kit according to the manufacturer’s protocol (Amaxa Inc, Gaithersburg, MD). The transfected cells were plated in 96-well black isoplate and cultured for 48 h after transfection. We used the Synergy H4 plate reader to monitor the BRET^2^ signal (Fluorescence/Luminescence ratio). The relative fluorescence (515/30 nm) and luminescence (410/80 nm) raw data were detected from each well after adding DeepBlue C (5 μmol·L^−1^) in the presence or absence of compound MS2 (10 μmol·L^−1^). In addition, based on the identification of crucial contact residues [e.g. Lys(K)^874^ and Arg(R)^877^ in PC2-CTT and Tyr(Y)^4236^ in PC1] that bind to MS2 in the computational modeling, we used a Q5 site-directed mutagenesis kit to generate amino-acid residue substitutions at the interaction sites in *wild-type* GFP2_PC2-CTT (K874E & R877P) and Rluc8_PC1-CTT (Y4236F) cDNAs to create mutant constructs (GFP2_PC2-CTT- K874E & R877P and Rluc8_PC1-CTT-Y4236F mutants) that disrupt the contact sites in MS2-PC1/PC2 binding pocket of *wild-type* PC1-CTT/PC2-CTT complex. Then we used the same approach to co-transfect GFP2_PC2-CTT-K874E & R877P mutant along with *wild-type* Rluc8_PC1-CTT or Rluc8_PC1-CTT-Y4236F mutant along with *wild-type* GFP2_PC2-CTT constructs into HEK293 cells to measure the changes of the BRET^2^ signal after treated with vehicle or compound MS2 (10 μmol·L^−1^).

### Synthesis of MS2

A MS analogue, MS2 was synthesized to provide preliminary structure-activity relationship information based on the computational model shown in Figure 6A.^2^ The compound has a purity of greater than 98% and their structures were authenticated by standard analytical chemistry analyses.

### Statistical analysis

We evaluated differences between two groups by unpaired t-test, multiple groups by one-way ANOVA with Turkey’s multiple comparison test. All values are expressed as means ± S.D. All computations were performed using a commercial biostatistics software (GraphPad Software Inc. La Jolla, CA).

### Supplementary information

Supplemental Material
