# Supplementary material for: Genetic interactions between polycystin-1 and Wwtr1 in osteoblasts define a novel mechanosensing mechanism regulating bone formation in mice
Source: Bone Res. 2023 Oct 26;11:57. doi: 10.1038/s41413-023-00295-4 (PMC10603112; doi:10.1038/s41413-023-00295-4)
Supplement: Supplementary file 1 — Supplemental Material [file 41413_2023_295_MOESM1_ESM.pdf]

Supplementary Information

Figure S1

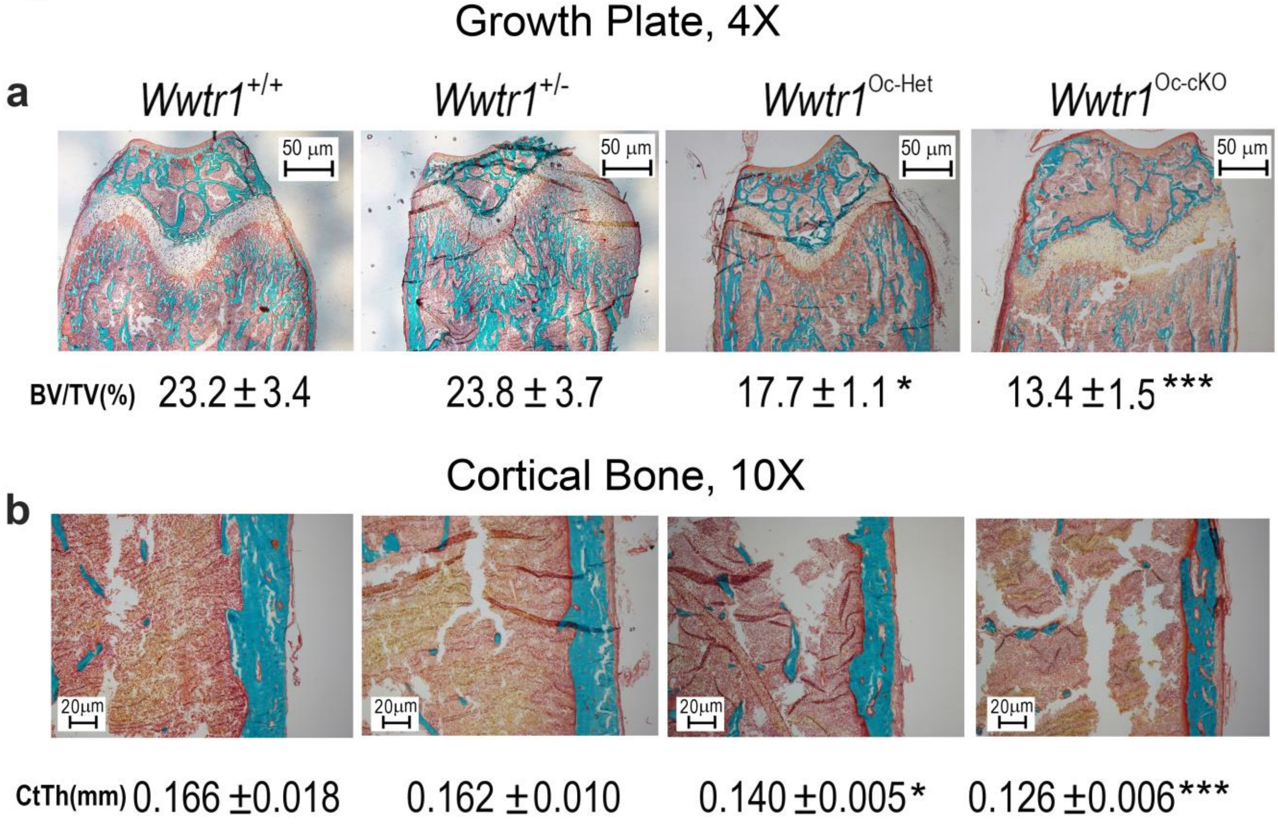

Supplementary Figure S1. Histological analysis of conditional *Wwtr1* deletion in bone. (**a** & **b**) Goldner staining of non-decalcified bone. Representative images of distal femur sections displayed a gene-dosage dependent reduction in trabecular bone volume under growth plate **(a)** and cortical thickness in diaphyseal area **(b)** from 8-week-old conditional *Wwtr1* deleted mice compared with age-matched control mice. Data are expressed as the mean ± S.D. from 6 individual mice (n=6). *P < 0.05, **P < 0.01, ***P < 0.001 compared with *wild-type* control mice. P values were determined by 1-way ANOVA with Tukey's multiple-comparisons test.

Figure S2

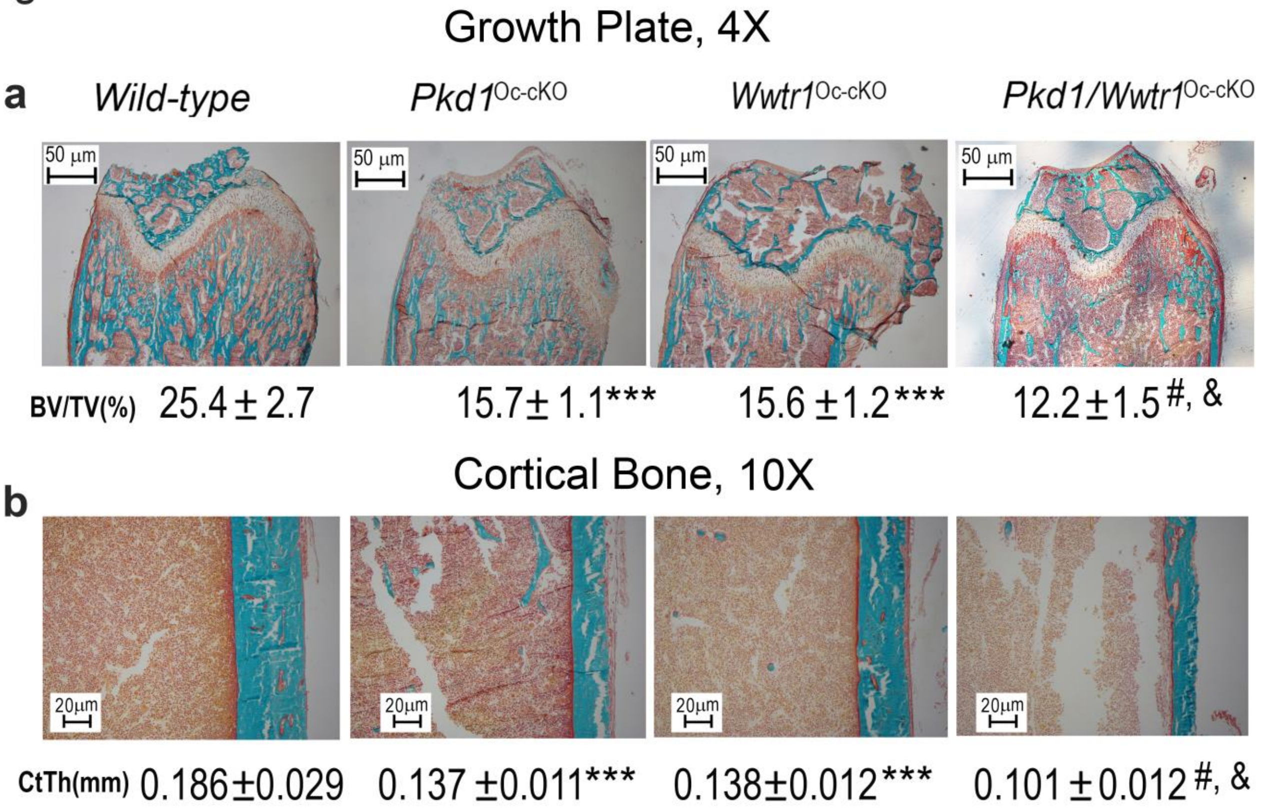

Supplementary Figure S2. Histological analysis of conditional *Pkd1* and/or *Wwtr1* deletion in bone. (**a** & **b)** Goldner staining of non-decalcified bone. Representative images of distal femur sections displayed significant reductions in trabecular bone volume under growth plate **(a)** and cortical thickness in diaphyseal area **(b)** from 8-week-old conditional *Pkd1* and/or *Wwtr1* deleted mice compared with age-matched control mice. Data are expressed as the mean ± S.D. from serum samples of individual mice (n=6). $^{*}P < 0.05$, $^{**}P < 0.01$, $^{***}P < 0.001$ compared with *wild-type* mice, $^{\#}P < 0.05$, $^{\#\#}P < 0.01$, $^{\#\#\#}P < 0.001$ compared with *Wwtr1*$^{Oc\text{-}cKO}$ mice, and $^{\&}P < 0.05$, $^{\&\&}P < 0.01$, $^{\&\&\&}P < 0.001$ compared with *Pkd1*$^{Oc\text{-}cKO}$ mice, respectively. *P* values were determined by 1-way ANOVA with Tukey's multiple-comparisons test.
